# The correlation between the electrode configuration and histopathology of irreversible electroporation ablations in prostate cancer patients

**DOI:** 10.1007/s00345-015-1661-x

**Published:** 2015-08-22

**Authors:** W. van den Bos, D. M. de Bruin, R. R. Jurhill, C. D. Savci-Heijink, B. G. Muller, I. M. Varkarakis, A. Skolarikos, P. J. Zondervan, M. P. Laguna-Pes, H. Wijkstra, T. M. de Reijke, J. J. M. C. H. de la Rosette

**Affiliations:** Department of Urology Academic Medical Center, University of Amsterdam, Amsterdam, The Netherlands; Department of Pathology, Academic Medical Center, University of Amsterdam, Meibergdreef 9, 1105 AZ Amsterdam, The Netherlands; Department of Biomedical Engineering and Physics. Academic Medical Center, University of Amsterdam, Amsterdam, The Netherlands; 2nd Department of Urology, Athens Medical University, University of Athens, Athens, Greece; Department of Electrical Engineering, Eindhoven University of Technology, Eindhoven, The Netherlands

**Keywords:** Irreversible electroporation, IRE, Prostate cancer, Planning, IRE, Focal therapy

## Abstract

**Purpose:**

Irreversible electroporation (IRE) is a novel minimally invasive therapy for prostate cancer using short electric pulses to ablate prostate tissue. The purpose of this study is to determine the IRE effects in prostate tissue and correlate electrode configuration with the histology of radical prostatectomy (RP) specimens. We hypothesize that the area within the electrode configuration is completely ablated and that the area within the electrode configuration is predictive for the ablated area after treatment.

**Methods:**

A prospective phase I/II study was conducted in 16 consecutive patients with histopathologically confirmed prostate cancer scheduled for RP. Focal or extended IRE treatment of the prostate was performed 4 weeks prior to RP. The locations of the electrodes were used to calculate the planned ablation zone. Following RP, the specimens were processed into whole-mount sections, histopathology (PA) was assessed and ablation zones were delineated. The area of the tissue alteration was determined by measuring the surface. The planned and the histological ablation zones were compared, analysed per individual patient and per protocol (focal vs. extended).

**Results:**

All cells within the electrode configuration were completely ablated and consisted only of necrotic and fibrotic tissue without leaving any viable cells. The histological ablation zone was always larger than the electrodes configuration (2.9 times larger for the 3 electrodes configuration and 2.5 times larger for the ≥4 electrode configuration). These ablation effects extended beyond the prostatic capsule in the neurovascular bundle in 13 out of 15 cases.

**Conclusions:**

IRE in prostate cancer results in completely ablated, sharply demarcated lesions with a histological ablation zone beyond the electrode configuration. No skip lesions were observed within the electrode configuration.

**Clinical trials:**

ClinicalTrials.gov Identifier: NCT01790451 https://clinicaltrials.gov/ct2/show/NCT01790451

## Introduction

Prostate cancer has become the most prevalent cancer in men and forms a significant health risk in the Western world [1]. PSA tests are currently performed regularly and imaging techniques have improved, leading to a considerably increased detection of localized prostate cancer [1]. The current treatment options for localized prostate cancer are active surveillance and therapy with curative intent (surgery or radiotherapy), depending on the stage of the tumour and patients’ consideration. A variety of ablative therapies has been introduced for treatment of localized prostate cancer. It functions as middle ground between the traditional options. The rationale behind these so-called focal therapies is to target only the tumourous areas and leave healthy tissue and adjacent structures intact. Most used ablative technologies are cryotherapy, high-intensity focused ultrasound (HIFU) and radiotherapy [2]. A novel technique in the armamentarium is irreversible electroporation (IRE). It uses pulsed high-voltage low-energy direct electric current for ablation. The electric energy is delivered through needle-electrodes, placed circumferential around the tumour zone. Cell-membrane potentials are disturbed by the consecutive high-voltage (usually around 1500 V/cm) electric pulse trains between at least two spatially separated electrodes, which leads to irreversible permeability of cell membranes and results in apoptotic cell death [3]. Literature on IRE reports advantages as sparing surrounding vital structures including blood vessels and connective tissue [4]. By sparing these structures unharmed, patients might maintain their potency and continence. Successfully treating prostate cancer requires an accurate prediction of the treatment zone through treatment planning. Planning of IRE is currently based on mathematical and numerical models in multicellular tissue models and animals studies [5–7]. The aim of this study was to assess the IRE ablation zone in specimens from radical prostatectomies by using different ablation protocols and to correlate the cross-sectional ablation zone on histopathology with the electrode configuration. We hypothesize that (1) the area within the electrode configuration is completely ablated; (2) the area within the electrode configuration is predictive for the cross-sectional ablated area after treatment, irrespective to the used ablation protocol.

## Methods

### Setting

Sixteen patients with confirmed localized prostate cancer, scheduled for a radical prostatectomy, were enrolled between August 2013 and April 2014 to undergo an IRE treatment approximately 4 weeks prior to RP. The patients’ mean age was 60 years (range 44–75) and had an average serum prostate specific antigen (PSA) concentration of 9.5 µg/L (range 4.4–22.5). Patient’s characteristics are shown in Table 1. Twelve patients were treated at the university hospital in Amsterdam and four patients in the university hospital in Athens. The institutional review boards of both hospitals approved the study. Written informed consent was obtained from every patient.

**Table 1:** Patient characteristics

	Patients (*N* = 16)
Age (years)	60 (range 44–75)
Serum PSA (µg/L)	9 (range 3.6–25)
Prostate volume (mL)	39 (range 19–60)
Gleason score (TRUS-guided biopsy)	
3 + 3	8
3 + 4	3
4 + 3	3
4 + 4	2
Disease distribution (TRUS-guided biopsy)	
Unilateral	11
Bilateral	5
Gleason score (RP specimen)	
No tumour present	1
3 + 3	7
3 + 4	6
4 + 3	1
3 + 5	1
4 + 4	0
Disease distribution (RP specimen)	
Unilateral	1
Bilateral	14
Tumour stage	
pT0	1
pT2c	11
pT3a	4

### IRE procedure

The IRE procedure was performed using transperineally inserted electrodes through a brachytherapy grid under continuous ultrasound guidance. The location of each electrode and the distances between the electrode pairs were assessed on ultrasound and used as prediction of the ablation zone.

**Fig. 1:**
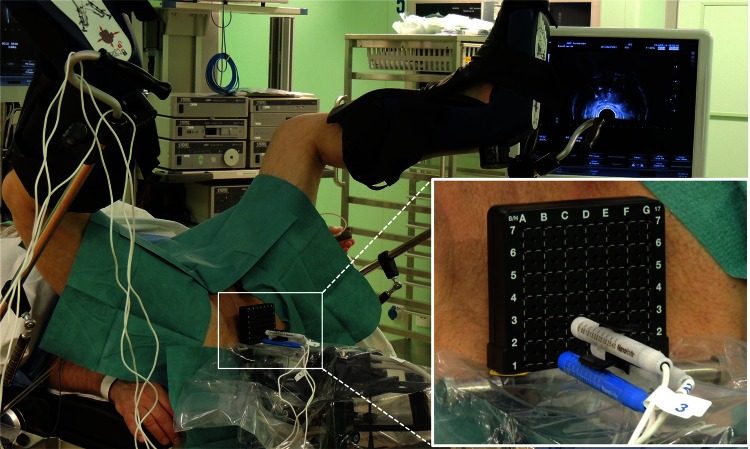
Patient in lithotomy position with three transperineally inserted electrodes under ultrasound guidance

The IRE treatment was performed using the Nanoknife^®^ (AngioDynamics Inc., Queensbury, NY) (Fig. 1). A low-energy direct current generator delivering high-voltage pulses by 19-gauge monopolar electrode needles. The procedures are performed under general anaesthesia. An electrocardiogram (ECG)-gating device (Accusync, Milford, Connecticut) was used to synchronize pulse delivery within the refractory period of the heart to prevent cardiac arrhythmias. Rocuronium was intravenously administered to achieve adequate muscle relaxation and was confirmed by twitch absence using a peripheral nerve stimulation test.

The IRE treatment was performed following a focal or extended ablation protocol. To achieve focal prostate ablation, one 2- and five 3-electrode configurations were performed. Ten extended ablations were performed using four or more electrodes. The active exposure length of the electrodes was set on 15 mm. The electrodes delivered 90 pulses of 90 μs duration each between every pair, with a pulse intensity set at 1500 V/cm (voltage-to-distance ratio). A current of 20–40 A runs during the pulse. The standard setting of 1500 V per cm distance between the needles was adapted when the current showed low amperage or high amperage, with a maximum of 3000 V. All electrodes were inserted at a distance of at least 5 mm from the rectal wall. In one patient, a pullback of 15 mm of all four electrodes was performed after the first ablation to increase the length of the ablation zone. During another procedure of a four-electrode configuration, the two lateral electrodes were repositioned after one ablation cycle to the other lobe, (covering the urethra) in order to perform a bilateral ablation. At this phase of research, the IRE treatment was not performed with curative intent. The electrodes were inserted in a lobe found positive by biopsy, however, exact targeting of the tumour was not pursued. The procedures and IRE device are described in detail in van den Bos et al. [8].

### Treatment prediction

**Fig. 2:**
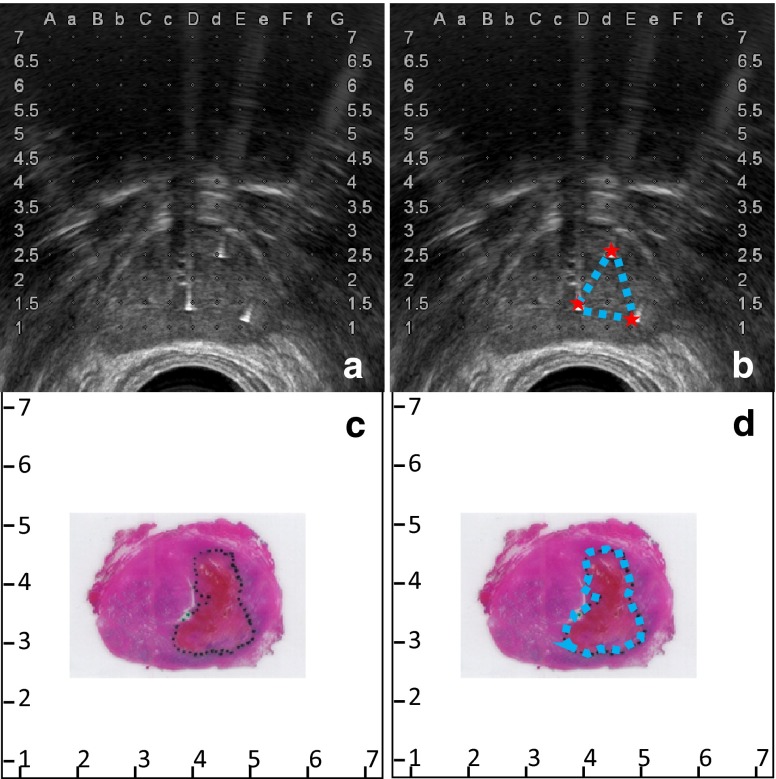
**a** Ultrasound image showing the three inserted electrodes. **b** The area within the electrode configuration is delineated. **c** H&E slide with the outlined ablation zone. **d** The ablation zone is delineated

**Fig. 3:**
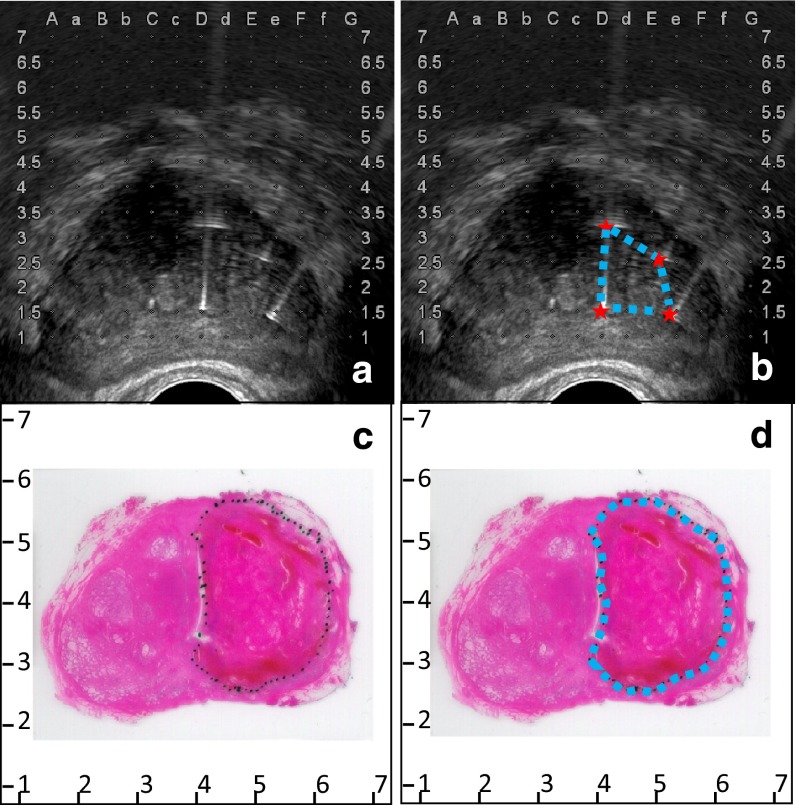
**a** Ultrasound image showing the four inserted electrodes. **b** The area within the electrode configuration is delineated. **c** H&E slide with the outlined ablation zone. **d** The ablation zone is delineated

The area within the electrode configuration was determined by the locations of the inserted electrodes as measured on the ultrasound images as indicated in Fig. 2a (focal ablation protocol) and in Fig. 3a (extended ablation protocol). The area within the configuration was delineated (Figs. 2b, 3b). Ultrasound imaging was scaled using the grid on the images, and the areas within the electrodes were determined using image analysis software ImageJ/FIJI. These areas were used as prediction of the ablation zone.

### Histology assessment

Macroscopic assessment and processing included measurement of size and weight, and orientation was achieved by using multiple colours for different planes. After formalin fixation the prostate was cut into serial sections of 3–5 mm thickness, perpendicularly to the urethra. The tissue was embedded in paraffin after which 4-µm-thick sections were cut and examined with an H&E stain. The characteristics of both the ablation zone (PA) and non-ablated zone were determined by light microscopy. The tissue is considered affected by IRE if the cells showed necrosis and/or denudation in case of epithelial tissue and necrosis and denudation are easily seen on an H&E slide. The margins of the ablation zone and tumour zones were delineated on the slides (Figs. 2c, d, 3c, d ). Furthermore, any affected essential structure (urethra, capsule, neurovascular bundle) was determined per patient and scored by the uropathologists. The correlation of the distance between electrodes and essential structures was measured in mm using ultrasound images. Because of non-normal distributed, non-paired data, statistical analysis was performed using a two-tailed Mann–Whitney test.

### Histology ablation areas

The edited slides were scanned 40X with the IntelliSite Ultra-Fast Scanner (Philips, Best, The Netherlands), and the areas of tissue alterations were determined by segmentation as designated by the pathologists. Subsequently, the slide representing the centre of the ablation zone, containing the most expanded ablation, was determined. The precisely outlined areas were scaled using the dimensions of the microscope slide. The ablation areas were calculated with the use of ImageJ/Fiji. The extracted cross-sectional ablation zones were adjusted for shrinkage during fixation using the pre-fixation prostate dimension divided by the post-fixation dimension.

### Electrode configuration and histology correlation

To assess whether the electrode configuration correlates to PA, all the areas within the inserted electrodes (in cm^2^) were directly compared and matched with the cross-sectional histology areas.

### Analysis of focal versus extended ablation zones

It was hypothesized that no difference would be assessed between the two ablation protocols in the comparison of the predicted and histological determined ablation areas. To test validity, the factorial difference between the predicted and histology area in cm^2^ is plotted for the two protocols by dividing histology analysis by the electrode configuration.

## Results

### IRE procedure

Six patients were treated using the focal ablation protocol. The extended protocol was performed in the remaining ten patients. All the procedures went uneventful, and no serious adverse events were observed during the procedure or hospital stay. None of the patients have been withdrawn because of adverse events. The voltage-to-distance ratios ranged from 1200 to 2100 V/cm with currents between 15 and 45 A. The procedure specifications per patient are shown in Table 2.

**Table 2:** Procedure specifications

Patient	Number of electrodes	Voltage-to-distance ratio (V/cm)	Range amperage (A)
1	2	1500	15–18
2	3	1500–1700	22–28
3	4	1500	21–42
4	4	1200–1500	22–45
5	3	1500–1800	26–35
6	4 + pullback	1350–1500	25–45
7	3	1500–1650	22–28
8	6	1350–1500	25–40
9	4	1500–1800	25–38
10	4	1500	23–35
11	3	1500–1800	21–31
12	4	1500–1650	32–42
13	4	1500–1800	20–40
14	4	1500–1950	21–30
15	3	1500–2100	18–25
16	3	1500	22–32

### Histology assessment

Gross examination showed sharply demarcated haemorrhagic lesions at the ablation site in all cases, except for the specimen of patient one where only two electrodes were used. Microscopic assessment showed haemorrhagic, necrotic and fibrotic areas in all cases, again except for patient one. In the first patient the specimen contained only fibrotic tissue with a bilateral extension, without any relation to the unilaterally inserted electrodes. This patient was the only one treated with two electrodes with a low current output (15–18 A), what is considered as insufficient by the manufacturer. Therefore, the case was excluded from further histological analysis. All other prostate specimens showed sharply delineated ablation zones with completely non-viable tissue consisting of necrotic and fibrotic tissue within the electrode configuration. The IRE effects comprised areas ranged from 5 to 40 % of the total prostate tissue.

### Electrode configuration–histology association

**Fig. 4:**
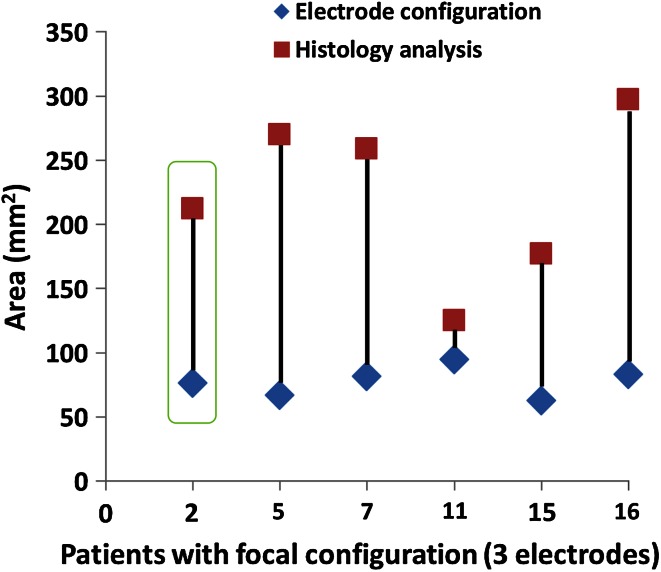
The areas within the electrode configuration and the cross-sectional areas of the histology analysis of the focal ablation protocol, presented per patient. The results outlined in *green* are from the same patient as displayed in *green* are from the same patient as displayed in Fig. 2

**Fig. 5:**
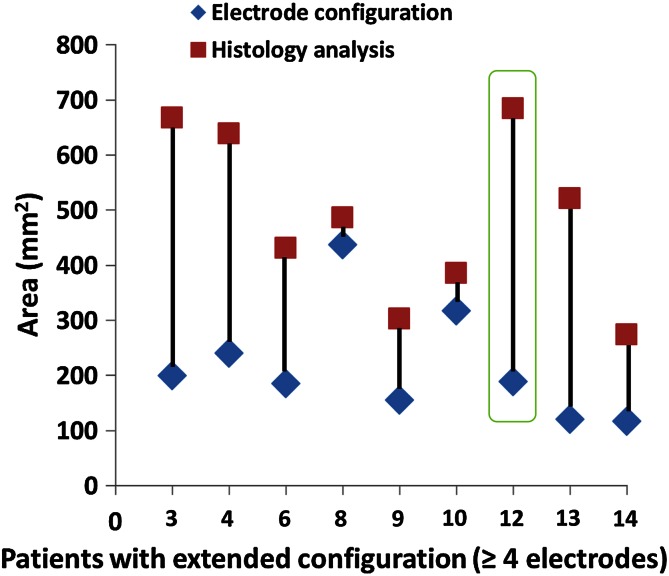
The areas within the electrode configuration and the cross-sectional areas of the histology analysis of the extended ablation protocol, presented per patient. The results outlined in *green* are from the same patient as displayed in Fig. 3

**Fig. 6:**
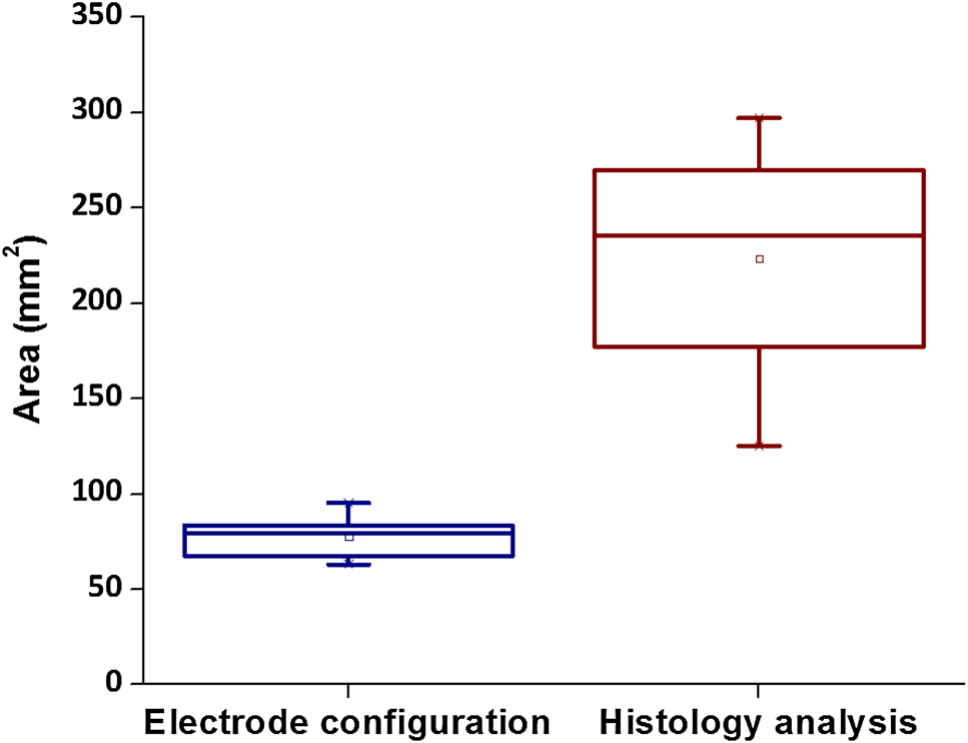
*Boxplots* showing the average results of the areas electrode configuration of the focal ablation protocol and the outcomes of the histology analysis

**Fig. 7:**
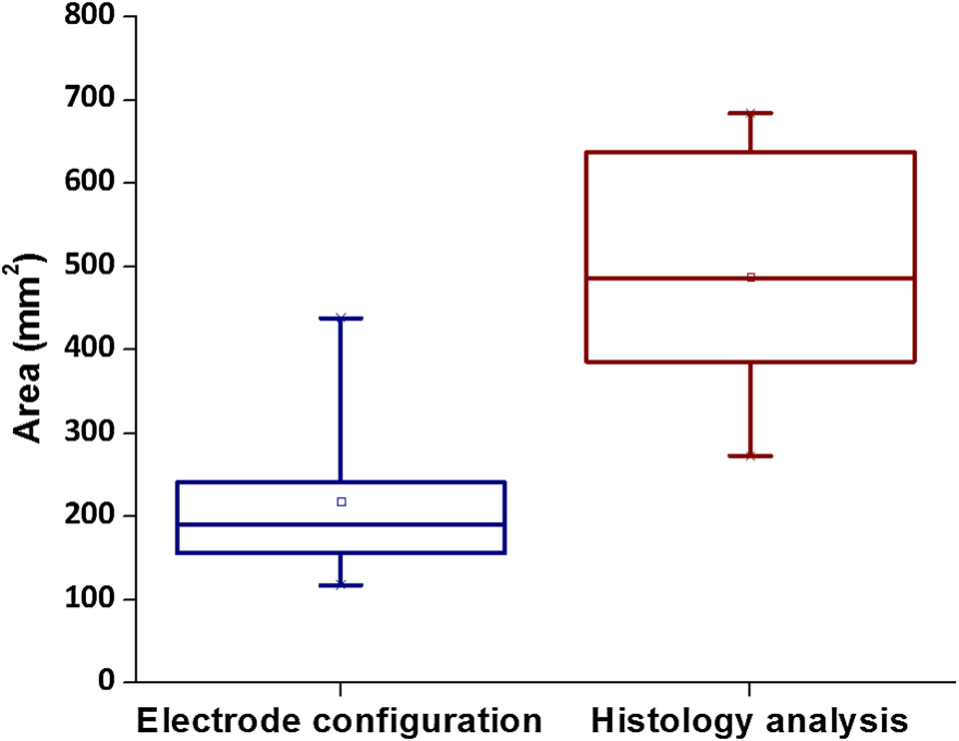
*Boxplots* showing the average results of the areas electrode configuration of the extended ablation protocol and the outcomes of the histology analysis

**Fig. 8:**
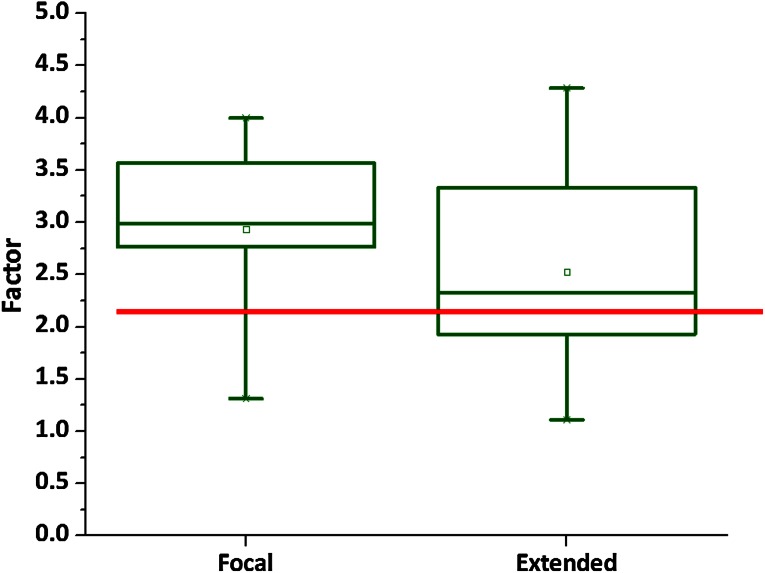
*Boxplots* showing the factorial outcomes between the areas of the electrode configuration and the histology analysis displayed per group. The *red line* represents the average of the factorial outcomes from the two ablation protocol

The areas traced within the electrode configuration (in mm^2^) were compared with the area on the H&E PA image and are displayed per patient in Fig. 4 for the focal ablation protocol and in Fig. 5 for the extended ablation protocol. The results, grouped per protocol, are shown in Figs. 6 and 7, respectively. None of the patients had skip lesions within the configuration of the electrodes. When using the focal ablation protocol, the histological analysis showed ablation zones of 2.9 times (range 1.3–4.0) greater than the area of the electrode configuration. Using the extended ablation protocol, the histological analysis showed ablation zones of 2.5 times (range 1.1–4.3) greater than the electrode configuration. Patient 4 in the focal protocol and patient 4 and 6 in the extended protocol appeared as outliers, with a relatively small ablation area. These variant outcomes could not be explained by the prostate tissue composition including the presence of glandular hyperplasia, inflammation, atrophy, Gleason score or prostate size. The factorial outcomes were presented per ablation protocol in Fig. 8. No significant difference was found between these outcomes.

**Fig. 9:**
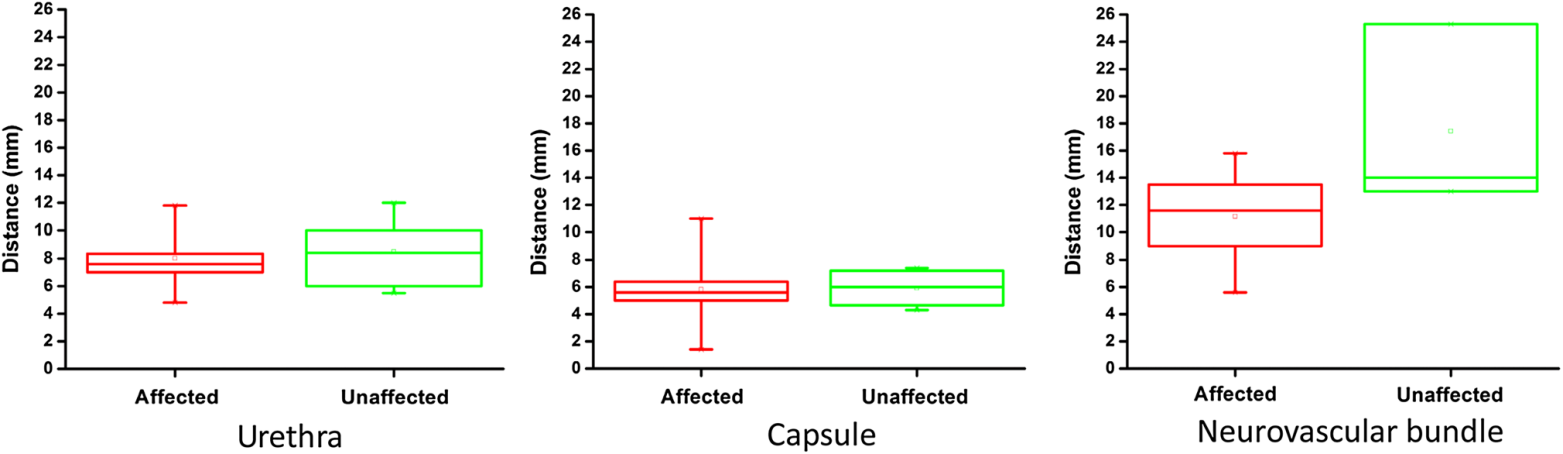
*Boxplots* showing of damaged and non-damaged essential structures revealing no difference between structures close to and more distant to the inserted electrodes (urethra *P* value 0.56, min–max = 4.8–12, prostatic capsule *P* value 0.94, min–max = 1.4–11, neurovascular bundle *P* value 0.58, min–max = 5.6–25.3)

IRE effects were observed extending beyond the prostatic capsule in twelve cases and in the neurovascular bundle in thirteen of the fifteen prostates. The urethra was affected due to IRE treatment in nine prostates. Chronic inflammation varied from mild to moderate with only one cases showing focal severe inflammation. Statistical analysis of affected versus unaffected tissue structures revealed no difference between structures close to or at distance to the inserted electrodes (urethra *P* value 0.47, IQR = 5.8–9.6, prostatic capsule *P* value 1.0 IQR = 5.0–6.4), neurovascular bundle *P* value = 0.38, IQR 4.9–6.3) as shown in Fig. 9.

## Discussion

The present study was designed to determine whether IRE was able to achieve complete cell death. It is crucial for ablative therapies to secure that the targeted zone is entirely ablated, without leaving any viable cells. In this study, the histology outcomes confirm that IRE effectively ablates all prostate tissue enclosed within the area of the inserted electrodes. The first examined viable cells were localized at the borders of the ablation zone. All inserted electrodes were placed with at least 5 mm distance to the rectal wall. During follow-up, no clinically significant adverse events concerning rectal wall damage occurred. The evaluation of the exact safe margin to be kept from the rectum was beyond the scope of this trial; however, in four instances a margin between 4.5 and 6.0 mm was kept with no subsequent adverse events.

Similar studies performing ablative therapies followed by RP show various results. Pisters et al. [9] did not achieve complete tumour destruction with whole-gland cryotherapy in three of the seven patients. Grampsas et al. [10] reported the potential for salvage radical prostatectomy in biopsy-proven recurrences of the 62 evaluated cases treated with complete cryoablation. Six of these patients underwent a salvage radical prostatectomy showing necrotic tissue in the area of probe-placement with a sharp border demarcating these regions from the adjacent viable oedematous connective tissue. The whole-mount sections of the prostate specimen revealed residual prostate cancer after supposed successful cryoablation. Two comparable studies were performed to evaluate the histological impact and efficacy of transrectal HIFU. Beerlage et al. [11] showed incomplete necrosis at the dorsal side of the prostate in all treated patients. In two of the nine cases, this area contained vital tumour tissue. Madersbacher et al. [12] achieved a successful ablation in three out of ten participating patients using HIFU. In the remaining seven patients, the tumour was partly targeted but persistent tumour tissue was found with histopathological examination. These studies showed that complete tissue destruction was not achieved in all participants. In contrast, our study using IRE ablation showed that within the targeted area all cells were destroyed. Several other papers, mostly based on canine studies, confirm our findings using the IRE technology [5], [12], [13]. However, the sizes of the ablation areas in histology are much larger than the configuration, with a factor of 2.7 on average. An explanation is that the ablation zone follows the electrical field (E-field) and this expands beyond the electrodes like described in the mathematical model of irreversible electroporation by Edd and Davalos [5]. Also potential thermal properties accompanying the IRE effects like described by Wagstaff et al. [13] may enlarge the ablation area.

Limitations of our study include possible shrinkage artefacts and the short term of follow-up of 4 weeks. During these 4 weeks following the IRE procedure, the inflammatory and fibrotic processes resulting in necrosis causes deformation of the prostate gland. An additional limitation is the small sample size of sixteen patients. Therefore, the results may not be generalised to all prostate cancer patients. A successive randomized clinical trial covering the next phase has already started under the umbrella of the Clinical Research Office of the Endourological Society (CROES) and aims to include 200 patients (NCT01835977). The study was also limited by the maximal follow-up of 4 weeks due to the need for RP in these patients. Although longer follow-up is desirable, postponing the scheduled RP was ethically no option. Actually, it is believed that the duration of the follow-up was sufficient since the histology showed necrotic and fibrotic elements implying the IRE effects were beyond the early effects of the treatment (inflammation) and before longer-term effects (fibrosis and repair). Interestingly, the first IRE treatment in our study was performed using a unilateral two-electrode configuration. Histological analysis did not show any necrotic area but diffuse bilateral fibrosis throughout the prostate. The reported current ranged from 15 to 18 A. Dunki-Jacobs et al. [14] stated that the change in resistance and the slope of the resistance should be used to assess successful tumour ablation during IRE in pancreas. The small change in current with an increase of 3 A of case 1 may imply an unsuccessful ablation. However, Neal et al. [15] performed 5 IRE treatments using two electrodes (1 in canine prostate and 4 in human prostate) generating low currents and high currents and the specimens all showed an ablation zones with a necrotic focus. Proper evaluation of an electrode configuration requires multiple repetitions. Since there was only one case with a two-electrode configuration and low current, the cause of the unsuccessful ablation remains unclear.

The findings of our study are important since it documents the targeted zone within the needles configuration where all cancer will be eradicated.

## Conclusion

Although IRE in prostate cancer results in a completely ablated, sharply demarcated ablation areas without leaving any viable cell within the electrode configuration. The electrode configuration using the focal ablation protocol and the extended ablation protocol, respectively, results in a 2.9 and 2.5 times greater cross-sectional histological ablation zone.
